# Revisiting the Economic Impacts of *Eimeria* and Its Control in European Intensive Broiler Systems With a Recursive Modeling Approach

**DOI:** 10.3389/fvets.2020.558182

**Published:** 2020-11-05

**Authors:** William Gilbert, Camille Bellet, Damer P. Blake, Fiona M. Tomley, Jonathan Rushton

**Affiliations:** ^1^Institute of Infection, Veterinary and Ecological Sciences, University of Liverpool, Liverpool, United Kingdom; ^2^Department of Pathobiology and Population Sciences, Royal Veterinary College, Hatfield, United Kingdom

**Keywords:** *Eimeria*, coccidiosis, broiler, disease control, model, economics, impact

## Abstract

Ionophore compounds active against *Eimeria* species are widely used in intensive broiler systems and have formed the backbone of coccidiosis control for almost 50 years. Producers, however, are under pressure to reduce ionophore use due to consumer concerns over antimicrobial usage in food animals, and antimicrobial resistance. Moreover, current vaccines against *Eimeria* are commonly considered to be less cost-effective in intensive broiler systems, especially in Europe where attenuated live vaccines are used. An economic assessment of the impact of *Eimeria* and the disease coccidiosis, including the cost implications of different efficacies of control, is therefore timely to provide evidence for industry and policy development. A mechanistic model of broiler production under varying infection and control states was used to construct a dataset from which system productivity can be measured. Coccidiosis impact increased rapidly as control efficacy decreased. In the total absence of control, median impact was found to maximize at between €2.55 and €2.97 in lost production per meter squared of broiler house over a 33 day growing period. Coccidiosis remains a major risk to intensive broiler systems and the model developed allows investigation of issues related to coccidiosis control, antimicrobial use and the development of antimicrobial resistance.

## Introduction

Coccidiosis is recognized as one of the principal intestinal disorders in intensive broiler production systems, limiting bird growth, and the efficiency of feed conversion (1). Caused by organisms of the genus *Eimeria*, there are three main species that account for most coccidiosis in broilers: *Eimeria acervulina, Eimeria tenella*, and *Eimeria maxima*. They are globally ubiquitous, with the most common of these, *Eimeria acervulina*, reported to have prevalence estimates in excess of 90% in some flocks (2–4). Furthermore, in addition to coccidiosis, *Eimeria* infection is a known risk factor for secondary infections and enteric dysbiosis, including necrotic enteritis (5, 6).

Infection occurs following the ingestion of sporulated oocysts from the environment, and proceeds through several phases of parasite asexual and sexual reproduction within epithelial cells of the digestive tract, after which the next generation of oocysts are excreted in the feces, where they sporulate to complete the life-cycle. The morbidity and mortality effects on the host are highly dependent on the infecting species, the infectious dose to which chickens are exposed (7) and the age at which infection first takes place (8). Induction of host protective immunity after recovering from infection varies considerably depending on the infecting species and the breed of chicken. In some cases, several cycles of infection and excretion may be required for full immunity to develop (9–11).

Ionophores are compounds that form complexes with specific ions and facilitate their transport across cell membranes. They are biologically active against both *Eimeria* and gram-positive bacterial species, are widely used in intensive broiler systems, and have been the backbone of coccidiosis control for almost 50 years (12). Field isolates of *Eimeria* species invariably have high levels of resistance to commonly used ionophores and also to many of the chemical coccidiostats (13–15), and it is very common for producers to use these different classes of drugs in rotation and shuttle programmes to minimize the impacts of drug resistance (16, 17). Interestingly, despite significant reductions in their efficacy, the ionophores continue to provide reasonable protection against clinical coccidiosis, and the accompanying growth and replication of resistant parasites, which can reach high levels in some flocks, allows for the host immune system to develop an effective and protective response (18).

Recent changes in society threaten to disrupt the status quo. Producers are under pressure to reduce the use of antimicrobial products in food animals, and the polyether ionophores, which are naturally produced by *Streptomyces* species, are included in this drive. While having no antimicrobial use in human health, they are classified as antibiotics in the United States of America and in 2018 more than 50% of total USA broiler production came from “antibiotic free” systems (19). This is reflected in a significant reduction in ionophore usage in the USA broiler industry, and a concomitant increase in the use of live coccidiosis vaccines (16). In Europe ionophores are classed as feed-additives, not antibiotics, nevertheless regulatory change affecting their use in livestock remains a distinct possibility (20). Any such policy decisions should to be supported by an evidence-based understanding of the impacts such reductions or bans on ionophore usage will have on food production systems. There is some evidence already emerging of the animal welfare consequences of removing certain critical antibiotics from intensive livestock systems (21), however the economic consequences are less clearly documented.

It is therefore opportune to assess current evidence on the impact of coccidiosis and the economics of its control in intensive broiler systems. In terms of economic impact, figures in the billions of dollars at the global level are often cited, extrapolated from the studies of Williams (1) in the United Kingdom. While much work in the past has been done to define coccidiosis impact under experimental conditions (22), assessing the impact of this disease from field data is complicated by many management, environmental (23, 24), and bird-level variables (25), necessitating large and detailed data sets. Although these kind of datasets exist in private companies, they are relatively inaccessible for reasons of commercial sensitivity. These facts, and the welfare implications of conducting *in vivo* experiments, have resulted in the development of *in silico* models to study coccidiosis in poultry systems.

The development of coccidiosis modeling has reflected the complexity of disease progression and pathogenesis. Parry et al. (26) described a recursive mathematical model of the *E. tenella* life cycle focused on tracking the development of immunity. Henken et al. (27, 28) took a similar recursive approach to assess the economic impact of differing levels of environmental contamination with *E. acervulina* on broiler production systems. Johnston et al. (29) expanded the modeling approach to *E. maxima* and *E. praecox*, focusing on the variation in replication rates of the parasite dependent on infectious dose. Further analysis by Klinkenberg and Heesterbeek (30, 31) explored the within host and between-host dynamics of *Eimeria* infection.

This paper presents an updated recursive model of *Eimeria* infection which permits the economic analysis of new developments in coccidiosis control. The model has application in the evidence-based assessment of policy and regulatory options, at a time when the use of antimicrobial products in food animal production systems is appearing increasingly unsustainable. With the specific focus on control, the model development was aimed to permit the following objectives:

1. To allow production parameters to be adjusted to reflect the different finishing and thinning weights found in intensive broiler systems.
2. To include models of infection for the three *Eimeria* species considered most common in intensive broiler systems: *E. maxima, E. acervulina*, and *E. tenella*.
3. To account for cumulative infection pressure, dose-dependent response to infection, and sub-clinical effects commonly observed in the field.
4. To incorporate immune dynamics at bird-level, allowing the simulation of vaccine-based control.
5. To allow control efficacy to be adjusted within and between production cycles for the investigation of shuttle and rotation programmes, drug resistance, and carry-over of infection between flocks.
6. To quantify the outcome of changes in coccidiosis control in economic terms at the level of the producer.

## Methods

### Overview

A simulation model of broiler production was constructed in R (32). Upon loading the model, the production cycle parameters must be defined by the user, who provides the model with desired finishing and thinning weights for the chickens, the area (m^2^) and number of broiler houses, and a maximum limit on stocking density (kg/m^2^). The model then calculates optimal stocking density of day old chicks, estimates thinning and end days for the production cycle, populates broiler houses, and proceeds to run at single-day time steps. As the model proceeds, birds feed, grow, and ingest *Eimeria* oocysts from the environment. Pathogen replication is simulated within each bird, and further oocysts excreted to the environment increase the environmental infection pressure. This pathogen replication is linked to a reduction in the efficiency of conversion of energy into growth of birds. As birds are exposed to *Eimeria*, immunity develops, replication becomes less efficient, and pathological action on the host diminishes. Each of these relationships is described in detail.

The critical assumptions made by the model with respect to the pathology and dynamics of infection are:

- Infectious oocysts are distributed in a homogenous manner within the environment.
- Oocyst ingestion likelihood increases with increasing feed intake, and increasing environmental concentration.
- The lifecycle of each *Eimeria* species following oocyst ingestion proceeds at fixed time intervals.
- Intracellular lifecycle stages of *Eimeria* cause cell damage in the host that produces the pathology associated with infection.
- Cell damage recovers after 8 days.
- Increasing the infectious dose of *Eimeria* produces more severe pathology.
- Each species of *Eimeria* has a defined maximal reproductive capacity in terms of oocysts excreted per oocyst ingested by the host.
- This reproductive capacity is determined by the product of reproductive rates across the asexual and sexual lifecycle stages of the pathogen.
- Reproductive capacity plateaus with increasing cell damage, while pathology increases in severity.
- Immunity is generated by the extracellular transition between intracellular lifecycle stages, on a 4-day time delay.
- Chemical or ionophore control of infection is based on a reduction in the rate of transition between intracellular lifecycle stages.

These assumptions, and how they are conceived within the model, are discussed in detail below.

### Bird Feeding and Growth

Published performance standards for as-hatched chicks, averaged across two well-known breeding lines (Ross 308 and Cobb 500) were obtained (33, 34). These data provide daily feed intake by mass, bodyweight gain, and total body weight in daily increments from hatching to ~70 days age/5.5 kg bodyweight according to a specified metabolizable energy (ME) feeding schedule. From these data, the following series of relationships were described by models fitted using Levenburg-Marquardt algorithm to recreate as-hatched performance standards:

- Bodyweight by age (days) (BW model)
- Daily feed intake by bodyweight (FI model)
- Weight gain by ME intake (Growth model)

Levenburg-Marquardt is an algorithmic process for fitting models to data by minimizing the sum of squares (35), and is the most widely applied method when a non-linear relationship has been specified (36). Model fitting was performed in R, using the *minpack.lm* package (37).

Laird-Gompertz growth curves have been used to describe chicken growth in the past (38–40), and a function of this form was applied here to describe the relationship between time and body weight (BW) in growing chickens. The BW model defines bodyweight *W*, at time *t* as a function of time and bodyweight at *t* = 0, and the constants ν and α such that:

$$\begin{array}{l} W_{t\;}=W_{0}\;e^{\left(\frac{\nu }{\alpha }\;\left(1-e^{-\alpha t}\right)\right)} \end{array}$$

This relationship is used within the model to predict the minimum age in days at which chickens attain target thinning and final weights in the absence of any impediment on growth. This sets the upper time limit of the model run. The maximum number of chickens in the house on thinning and end days is then calculated and the required thinning proportion to allow the stocking density to remain within the upper bound is calculated automatically, accommodating for a user-defined expected mortality rate.

Birds are placed in the house as day old chicks of mass 0.0565 kg, and feeding commences at daily increments. The FI model relates feed consumption as a function of the bird's current body weight, following the form:

$$\begin{array}{l} I_{t\;}=\rho +\tau \left(1-e^{-\sigma W_{t}}\right) \end{array}$$

The intake of feed *I* at time *t* follows a decreasing exponential of bodyweight *W* at time *t* at rate –σ. The constant ρ improved the fit of the curve at initial values, while τ represents the maximal asymptotic value of *I*. Following from this, the model calculates the daily potential for growth of the bird in response to feed intake by referring back to the feeding schedule. The model for growth follows that of Zuidhof et al. (41), where growth is a function of metabolizable energy intake:

$$\begin{array}{l} G_{t}=\frac{(1-L)(I_{t}\;\times \;ME_{t})-\delta W_{t}^{\varepsilon }-RFI}{\vartheta } \end{array}$$

Where *G* is weight gain at time *t, ME* is the metabolizable energy content of the feedstuff at time *t*, $\delta W_{t}^{\varepsilon }$ is the maintenance energy requirement for a bird of given body mass (*W*) at time *t, RFI* (the residual feed intake), δ, ε, and ϑ are constants. To relate infection status to growth rate, L is a variable coefficient representing the malabsorption of nutrients caused by *Eimeria* infection. The model records the total feed consumption at flock level, and the total output mass of chickens at thinning and finishing time. The parameter values applied for the model are given in Table 1. Feedstuff was scheduled according to the recommendations of the breeding companies, with starter feed on days 1–10, grower feed on days 11–24, and finisher feed on days 25+. The ME content was defined as 3,000, 3,100, and 3,200 kcal/kg for these three feed types, respectively.

**Table 1:** Parameter values for bird growth and feed consumption models.

**Parameter**	**Value**	**References**
δ	200.079	Model fit
*RFI*	−44.63	
**ϑ**	3146.14	
ε	0.75	(41, 42)
ρ	0.013	Model fit
τ	0.258	
σ	0.552	
α	0.045	
ν	0.224	

### *Eimeria* Lifecycle

**Figure 1:**
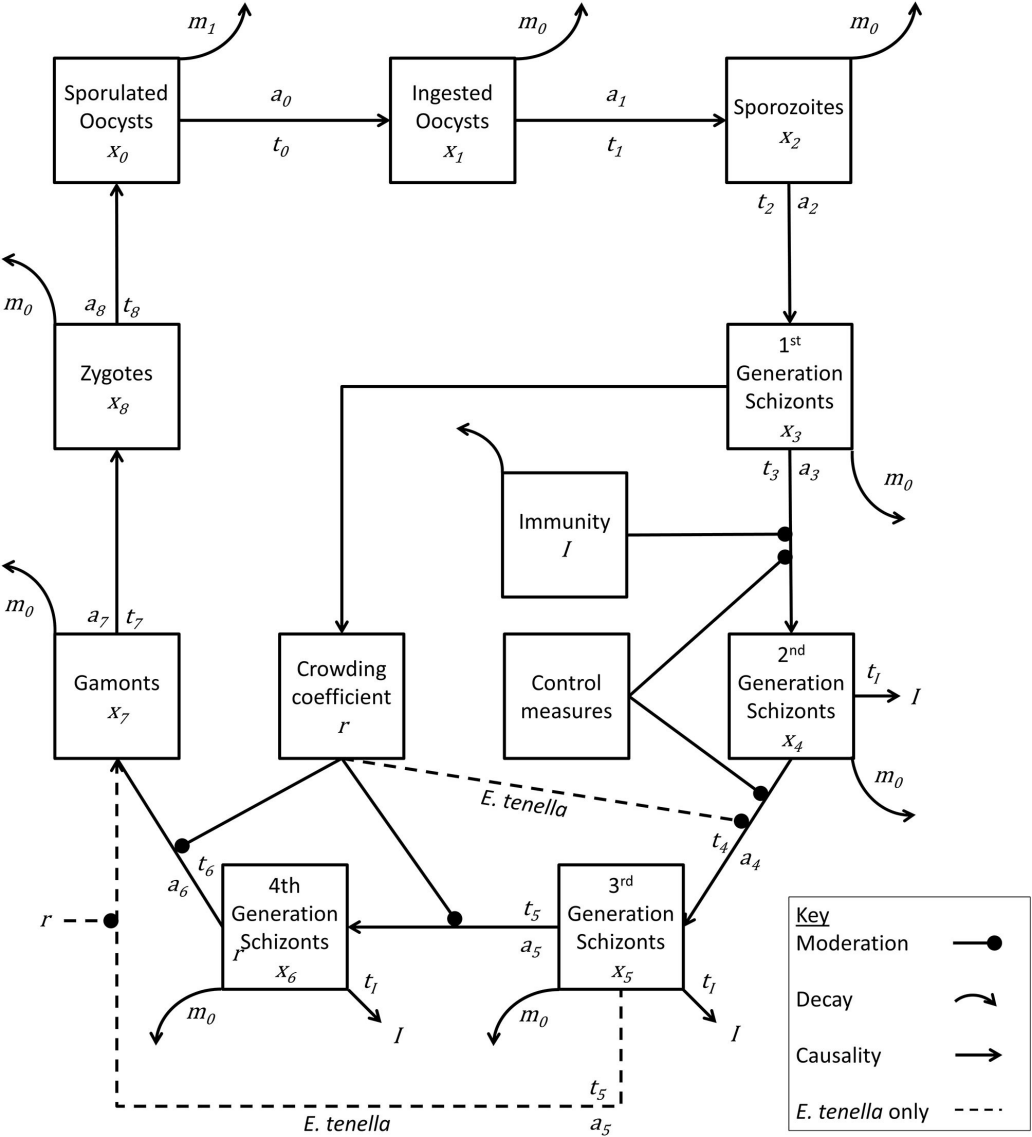
Lifecycle of *Eimeria* species. Life cycle stages are denoted by x_n_. Rate of advancement between stages is determined by coefficient a_n_. The time lag between each stage is represented by t_n_. Removal of pathogen from the system is indicated by m_n_. Immunity generation is indicated by *I. Eimeria tenella* has one fewer lifecycle stages, and proceeds directly from *x*_5_ to *x*_7_.

The model *Eimeria* lifecycle (Figure 1) followed the foundational structure developed by other authors (26, 28–30). In short, a recursive system of calculations operating at one-day time steps estimates daily change in pathogen lifecycle stages (*x*) in a deterministic manner at the level of the chicken and the level of the house, where each species of *Eimeria* has a number of developmental stages (*i*). The time interval in days between lifecycle stages (*t*_*i*_), the replication rate between stages (*a*_*i*_), and daily natural pathogen mortality (*m*) is specified for each lifecycle stage and for each species of *Eimeria*.

While detailed accounts of such models can be obtained within the literature, essentially the number of new *Eimeria* of life cycle stage *x*_*i*_at time *t* can be calculated from the number at stage *x*_*i*−1_ at time *(t-t*_*i*−1_*)*. Similarly, the number of *Eimeria* of life cycle stage *x*_*i*_leaving the lifecycle stage at time *t* can be deduced from the number new *x*_*i*_ at time *(t-t*_*i*_*)*, therefore recording a net change in each compartment at each time step:

$$\begin{array}{l} \frac{\partial x_{i}}{\partial t}=\;New\;\left(x_{it}\right)-\;New\;\left(x_{i\left(t-\;t_{i}\right)}\right) \end{array}$$

Establishing a lifecycle framework for each species of *Eimeria* allowed the interpretation of published experimental results using a deterministic structure where the dynamics and pathology of infection are interpreted with reference to the passage of the pathogenic organism through its lifecycle. Such experimental studies include investigations of single dose and repeated dose effects on weight gain and mortality, within-pen transmission rates, immunogenicity of single dose and repeated dose infection, and diminishing returns on increasing single-dose infections. These relationships are described in detail below.

The baseline replication rate for each species of *Eimeria* was estimated from experimental and microscopic studies (43–48). These maximum replication rates were modified to fit dose-dependent oocyst output rates, the “crowding effect,” shown in the results published by Williams (7). The pathogen lifecycle was modeled as a deterministic process in both time and replication rate such that oocyst excretion occurred at the time of peak excretion as observed *in vivo* (7, 49).

At each time step, environmental oocysts are inactivated at rate *m*_1_, and within-host lifecycle stages suffer a mortality rate of *m*_0_. All lifecycle parameters for *Eimeria* spp. are shown in Table 2.

**Table 2:** Lifecycle parameters for three *Eimeria* species, with reference sources.

**Parameter**	***Eimeria acervulina***	***Eimeria maxima***	***Eimeria tenella***	**References**
a_0_	α {oocyst concentration, feed intake}			
a_1_	8	8	8	(43–48)
a_2_	1	1	1	
a_3_	16	24	100	
a_4_	16	12	160	
a_5_	16	12	20	
a_6_	12	12	N/A	
a_7_	0.95	0.95	0.95	(50)
a_8_	0.7	0.7	0.7	(26)
t_0_	Variable with ingestion rate			Model fit to data (7, 49)
t_1_	0 days	0 days	0 days	
t_2_	1 day	1 day	1 day	
t_3_	1 day	1 day	1 day	
t_4_	1 day	1 day	1 day	
t_5_	1 day	1 day	2 days	
t_6_	1 day	1 day	N/A	
t_7_	0 days	1 day	1 day	
t_8_	0 days	0 days	2 days	
m_0_	0.08	0.08	0.08	(27)
m_1_	0.11	0.11	0.11	(26, 51)

Previous studies investigating total oocyst production and the pathogenic consequences of *Eimeria* infection have illustrated that while increasing the inoculation dose of oocysts increases the pathogenic action of the parasite, and initially increased the output of new oocysts from the host. A “crowding” threshold can be reached, however, at which point oocyst production plateaus or is diminished (7). The explanation for this phenomenon is not well-understood, appearing to result from an interaction of host cell availability, immune response and other factors (7, 29). In order to accommodate these two effects, an increase in pathogenicity with increasing pathogen load, whilst simultaneously inhibiting pathogen reproduction above a certain threshold, the crowding effect was emulated by an additional scaling coefficient in the reproduction rates of the final intracellular lifecycle stages for each species of *Eimeria*. In effect, this variable coefficient simulated a lack of host cell availability by taking into consideration the infection history of the individual bird. The number of first generation schizonts over time period *h* was used to define infection history. The duration of *h* was the duration of a complete cycle of intracellular infection summed with the time for cell repair, essentially a duration over which cell damage could be measured at time *t*. The number of new *x*_3_ schizonts formed in this period was used as a measure for the infection history to provide a historic exposure variable *x*_*E*_. For each species of *Eimeria*, the crowding coefficient was calculated to fit the reproductive rates estimated by Williams (7). For these results, *x*_*E*_was back calculated from the experimental protocol, and plotted against the reduction in replication rate observed. Following selection of a functional form to fit the observed curves, parameters were estimated by non-linear regression. The replication coefficient at *t* was then calculated dynamically within the new model first by:

$$\begin{array}{l} x_{E}=\;\overset{t}{\underset{t-h}{\sum }}x_{3} \end{array}$$

And then:

$$\begin{array}{l} r=\sqrt{\frac{r_{\text{max}}}{1+e^{-k\left(Logx_{E}\;-\;E_{0}\right)}}} \end{array}$$

Where *r*_*max*_ is the maximal value of *r, k* is a rate constant and *E*_0_ is the value of *Logx*_*E*_ at the inflection point. Parameter values are listed in Table 3.

**Table 3:** Parameters for pathology, immunity and replication for each *Eimeria* species modeled.

	**Parameter**	***E. tenella***	***E. acervulina***	***E. maxima***	**References/** **data source**
Pathology	β	2.295	0.434	0.716	Model fit (52)
	γ	18.879	21.058	19.942	Model fit (52, 53)
	η	0.947	0.947	0.947	(31)
Immunity	κ	0.000037	1 × 10^−7^	0.001896	Model fit (9–11, 54)
	ζ	0.00013	0.000191	0.000034	
	*a*	5.606	2.568	10.000	
	*b*	0.575	0.316	0.882	
	*c*	170,999	16.379	62.609	
	*t_*Y*_*	4	4	4	(55)
Replication	*r_*max*_*	0.158	0.069	0.212	(7)
	*k*	−1.153	−0.758	−0.775	
	*E_0_*	10.107	12.860	8.541	
Infection	Litter portion	2.66 × 10^−6^ kg			
	Litter intake	Triangular (0, 0.005, 0.03)			

### Infection

An environmental seeding rate for *Eimeria* oocysts of each species is provided to the model at the initiation of the model run. This is defined as oocysts per meter squared of broiler house. The rate at which chickens ingest environmental oocysts was back-calculated from the mean of results observed in floor-pen transmission studies by Velkers et al. (49). These results were adapted to fit with the pathogen replication model already described. First total oocyst excretion observed was placed within a deterministic time structure by assuming all oocysts are excreted on the day of peak excretion. From this, the timing and quantity of infectious dose were calculated from oocyst excretion results, using the pathogen replication model described above. With floor space and litter mass recorded within the experimental protocol, it was assumed oocysts were distributed homogenously within the litter mass, and thus a quantity of litter equal to that containing the infectious oocyst dose was ingested. This calculation led to the division of the litter into a number of “portions” that can be ingested by the broilers. A single litter “portion” was estimated to be 2.66 × 10^−6^ kg. The rate at which these portions are ingested was then calculated as a proportion of total feed intake, such that a dimensionless variable for litter as proportion of total diet was defined as a triangular distribution with minimum at zero, modal value at 0.005 and maximum at 0.03.

At each time step in the model, the total number of sporulated oocysts in the broiler house is divided by the total number of litter portions remaining to estimate the oocyst concentration per portion in the environment. Each bird draws from the triangular distribution and this number is multiplied by the bird's feed intake for the day to obtain a mass of litter consumed. This number is then divided by the portion size and rounded to the nearest integer to provide a number of portions consumed (*n*). Bird oocyst ingestion is then estimated as the sum of *n* samples from a Poisson distribution where λ is the expected oocyst count per portion. This process was repeated for each species of *Eimeria* present in the environment.

### Pathogenesis

Pathogenesis was modeled as arising from intracellular lifecycle stages (*x*_3_ to *x*_7_) of pathogen replication, which it was assumed resulted in lesion formation and malabsorption of nutrients from the gut lumen (56, 57). Malabsorption was represented by an additional variable coefficient (*L*) added to the growth model.

$$\begin{array}{l} L=\;\frac{1}{1+e^{-\beta \left(Logx_{s}\;-\;\gamma \right)}} \end{array}$$

Where x_s_ at time *t* is the sum of intracellular lifecycle stages (*x*_3_ to *x*_7_) between *t*- 8 and *t*, representing an 8-day period taken for the gut to heal.

$$\begin{array}{l} x_{s}=\;\overset{t}{\underset{t-8}{\sum }}\overset{7}{\underset{i=3}{\sum }}x_{it} \end{array}$$

The model for each species was fitted by Levenburg-Marquardt algorithm to the mean of experimental results published by Conway et al. (52) who present dose-dependent response to infection for each species by measuring changes in bodyweight gain at 7 days post-infection. The proportion of growth lost relative to uninfected controls over a 7-day experimental period described by Conway was assumed to be derived from the bird growth and pathogen replication models already estimated above, allowing a relationship between infection status and growth to be defined. Since each of these species of *Eimeria* inhabit different regions of the gut, it was considered likely that coinfecting species would not compete directly with one another and were therefore likely to produce a cumulative impact on weight gain. In the absence of significant volumes of literature on coinfection effects, response was assumed simply to be additive when simultaneous infection with multiple species occurs.

### Immunity

Immunity *(Y)* develops following the model proposed by Klinkenberg and Heesterbeek (30). The constants κ and ζ moderate the amount of new and proliferative immunity, respectively, subject to a time delay (*t*_*Y*_), while existing immunity decays at rate η. The values for these constants estimated by Klinkenberg and Heesterbeek were modified to reflect the different pathogen replication rates assumed within the model here. Immunogenicity was derived from the transitions between stages *x*_3_ and x_6_ inclusive. Grouped together these are termed *x*_*Y*_. The effect on immunity is delayed and occurs at *(t* + *t*_*Y*_*)*.

$$\begin{array}{l} x_{Y}=\sum_{i=4}^{6}New(x_{i(t-t_{Y})}) \end{array}$$

For each species of *Eimeria*, immunity is independent. Immune level is a recursive calculation:

$$\begin{array}{l} Y_{t}=\;\eta Y_{t-1}+\kappa x_{Y}+\;\zeta x_{Y}Y_{t-1} \end{array}$$

This formula allows the generation of immunity following single or repeat exposure to pathogen to be expressed quantitatively as a single variable.

To translate this variable into action, a function *f(Y)* was derived for each species of *Eimeria*. A collection of published experimental results (9–11, 54) provided the data for this estimation. For each published experimental protocol (*Eimeria* species, dose schedule), *Y* was calculated, as well as the level of inhibition of pathogen replication, measured as reduction in fecal oocyst count. This relationship was visualized and observed to approximate an asymmetric sigmoidal curve. A five-parameter logistic function with asymptotes at zero and one provided a form that was defined for each *Eimeria* species by non-linear least squares. This functional form is used when an asymmetric dose-response relationship is observed (58, 59).

$$\begin{array}{l} f\left(Y\right)=\;\frac{1}{1+{\left({\left(\frac{Y}{c}\right)}^{b}\right)}^{a}} \end{array}$$

Host immunity effect on the pathogen was expressed as a moderating coefficient in the transition of stages x_3_ to x_4_. Pathogenicity and immunity parameters are listed in Table 3.

### Control

To establish a baseline scenario for coccidiosis control, commonly applied ionophore and combination (ionophore plus chemical) coccidiostats were reviewed for their means of activity against *Eimeria* lifecycle stages. Nicarbazin-Narasin prevents the formation of sporozoites and merozoites. Salinomycin is shown to attack the extracellular merozoite stages of the pathogen lifecycle while sporozoites may be sufficiently resistant to allow host-cell invasion (60), although this may depend on the concentration of salinomycin to which the parasite is exposed and the exposure time (61, 62). In the model therefore, the action of control was simulated by assigning a control efficiency value *(C)* ranging between 0 and 1. Control action was divided between the development of new *x*_4_ and *x*_5_ generations by multiplying by (1-√*C*). This allowed the model to simulate the suppression of clinical disease by limiting intracellular lifecycle stages and reduction in oocyst excretion but also allowing immunity to develop.

The new oocyst formation functions in each compartment for *E. acervulina* and *E. maxima* are summarized:

$$\begin{array}{l} New\left(x_{it}\right)\{\begin{array}{c} x_{8\;\left(t-t_{8}\right)}\;\times \;a_{8}\;\times \;\left(1-m_{0}^{t_{8}}\right),\;i=0\\ \propto \{environmental\;oocyst\;concentration,\;feed\;intake\}\;i=1\\ x_{1\;\left(t-t_{1}\right)}\;\times \;a_{1}\;\times \;\left(1-m_{0}^{t_{1}}\right),\;i=2\\ x_{2\;\left(t-t_{2}\right)}\;\times \;a_{2}\;\times \;\left(1-m_{0}^{t_{2}}\right),\;i=3\\ x_{3\;\left(t-t_{3}\right)}\;\times \;a_{3}\;\times \;\left(1-m_{0}^{t_{3}}\right)\;\times \;\left(1\;-\;\sqrt{C}\right)\;\times \;(1-f\left(Y\right)),\;i=4\\ x_{4\;\left(t-t_{4}\right)}\;\times \;a_{4}\;\times \;\left(1-m_{0}^{t_{4}}\right)\;\times \;\left(1\;-\;\sqrt{C}\right),\;i=5\;\\ x_{5\;\left(t-t_{5}\right)}\;\times \;a_{5}\;\times \;\left(1-m_{0}^{t_{5}}\right)\;\times \;r,\;i=6\;\\ x_{6\;\left(t-t_{6}\right)}\;\times \;a_{6}\;\times \;\left(1-m_{0}^{t_{6}}\right)\;\times \;r,\;i=7\;\\ x_{7\;\left(t-t_{7}\right)}\;\times \;a_{7}\;\times \;\left(1-m_{0}^{t_{7}}\right),\;i=8\\ \; \end{array} \end{array}$$

And for *E. tenella* one fewer schizont stages:

$$\begin{array}{l} New\left(x_{it}\right)\{\begin{array}{c} x_{8\;\left(t-t_{8}\right)}\;\times \;a_{8}\;\times \;\left(1-m_{0}^{t_{8}}\right),\;i=0\\ \propto \{environmental\;oocyst\;concentration,\;feed\;intake\}\;i=1\\ x_{1\;\left(t-t_{1}\right)}\;\times \;a_{1}\;\times \;\left(1-m_{0}^{t_{1}}\right),\;i=2\\ x_{2\;\left(t-t_{2}\right)}\;\times \;a_{2}\;\times \;\left(1-m_{0}^{t_{2}}\right),\;i=3\\ x_{3\;\left(t-t_{3}\right)}\;\times \;a_{3}\;\times \;\left(1-m_{0}^{t_{3}}\right)\;\times \;\left(1\;-\;\sqrt{C}\right)\;\times \;(1-f\left(Y\right)),\;i=4\\ x_{4\;\left(t-t_{4}\right)}\;\times \;a_{4}\;\times \;\left(1-m_{0}^{t_{4}}\right)\;\times \;\left(1\;-\;\sqrt{C}\right)\;\times \;r,\;i=5\\ x_{5\;\left(t-t_{5}\right)}\;\times \;a_{5}\;\times \;\left(1-m_{0}^{t_{5}}\right)\;\times \;r,\;i=7\\ x_{7\;\left(t-t_{7}\right)}\;\times \;a_{7}\;\times \;\left(1-m_{0}^{t_{7}}\right),\;i=8\; \end{array} \end{array}$$

### Production and Economic Parameters

On running, the model records bird weight, mortality, and feed consumption per day over the course of the production cycle. On the estimated finish date, the total mass of extant chickens is recorded as system output, as is the total feed consumed. These data provide a point at which economic analysis of the production system can be performed.

To test the application of the model, production parameters exemplifying a typical European broiler-production system were selected and run through the model (Table 4). The production parameters were devised with the collaboration of European national producer associations who wished to remain anonymous. With data on precise input costs being commercially sensitive, a range of prices was applied to quantify producer margins over feed costs, expressed as Euros (€) per meter-squared.

**Table 4:** Production and economic parameters used to simulate intensive broiler production, selected to be representative of a typical European system.

**Production parameter**	**Value**	**Unit**
Finishing target weight	1.9	kg
Finish time	33	Days
Thinning of flock	No	–
Thinning weight	N/A	kg
House size	1,200	m^2^
Stocking density limit	42	kg/m^2^
Chick placements	27,714	chicks
Base mortality rate	4.2	%
Condemnation rate	1.08	%
Control schedule	1–33	Days
Feed price	270–320	€/ton
Output price	0.82–0.9	€/kg
Starter ration	3,000	kcal/kg
Grower ration	3,100	kcal/kg
Finisher ration	3,200	kcal/kg
Control efficacy	0–100	%

In the absence of data from the field on environmental oocyst concentrations at the beginning of the production cycle, the model was tested with fixed initial oocyst concentrations. To introduce disease into the system, the model was run 350 times with starting levels of *Eimeria* spp. oocysts from 0 to 5,000/m^2^ (0, 50, 500, 50,00) for each species. Given that the efficacy of control measures in the field is known to be variable, but of uncertain distribution, control efficacy was fixed at a range of levels across the distribution from 0 to 100% (0, 25, 50, 75, 100). Each model run simulated 1,200m^2^ of floor space, equivalent to 27,700 birds. Across 350 model runs, this generated a total simulation of 420,000 m^2^ of floor space and 9.7 m chickens.

## Results

**Figure 2:**
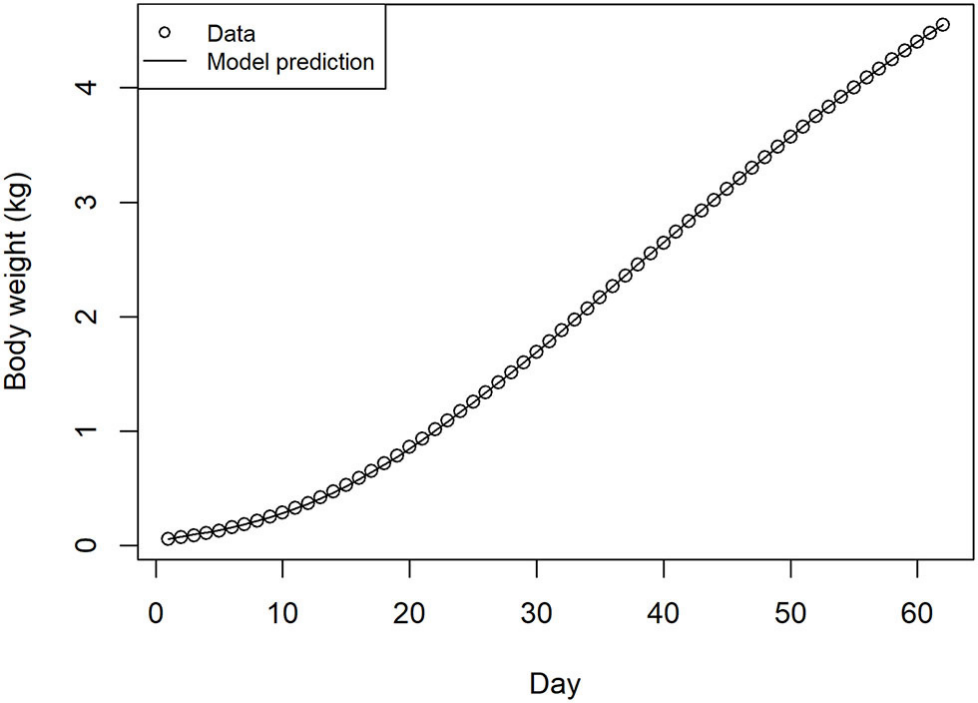
Source data and recursive growth model fit for bird weight over days of production, estimated from feed intake, feed schedule, and energy conversion models.

The fit of the model simulation for broiler growth in the absence of infection was found to be extremely close to the source data when tested to day 63, far beyond the finishing age of commercial flocks typical in Europe (Figure 2). This output was produced from the sequential application of the feed intake model, the metabolizable energy content of the feed determined from the feed schedule, and the broiler growth model.

**Figure 3:**
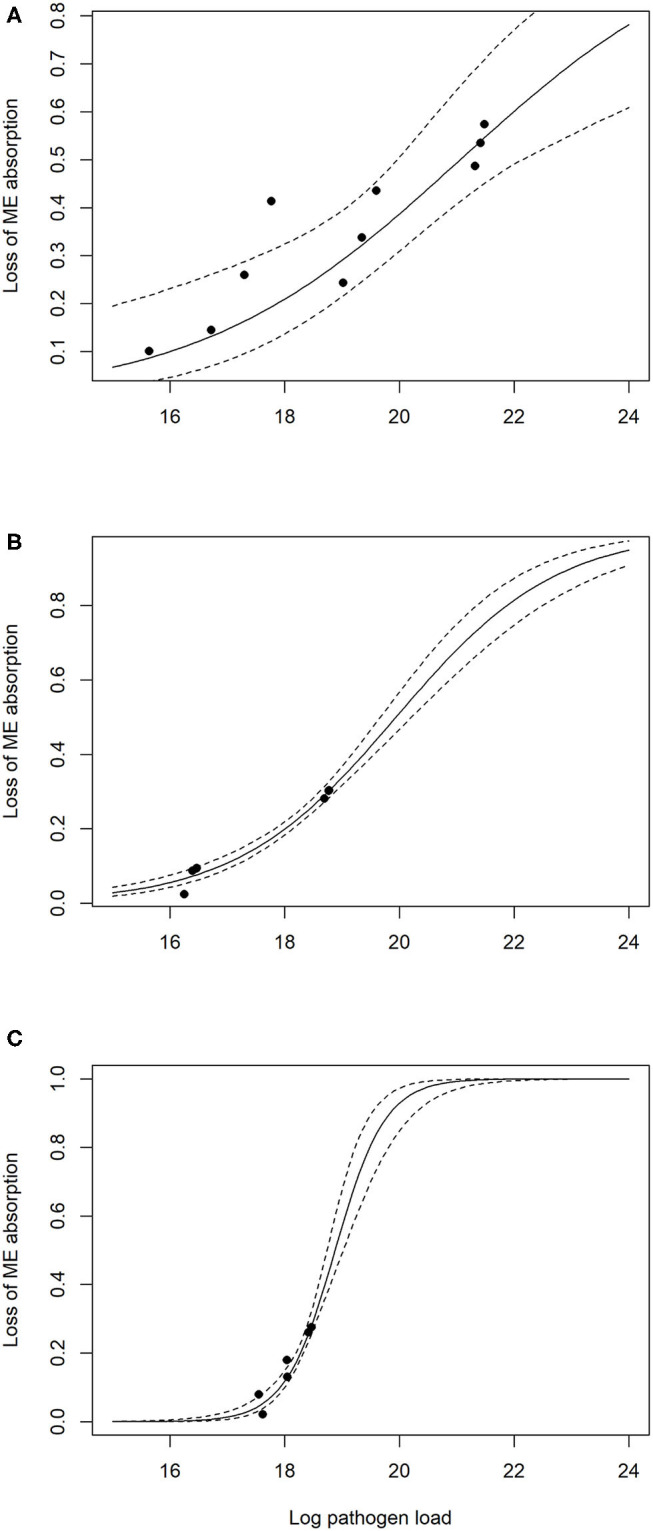
Pathogen influence on reduction in metabolizable energy (ME) absorption from the gut, lines show the mean and 95% CI of model prediction, with source data indicated by point: **(A)** *Eimeria acervulina*, **(B)** *Eimeria maxima*, **(C)** *Eimeria tenella*.

The output for the model of pathogenesis, shown as the reduction of absorption of metabolizable energy following infection for each of the three species of *Eimeria*, with reference points for source data, is presented in Figure 3. As expected, *Eimeria acervulina* shows the slowest increase in pathogenicity with increasing infectious dose, while *E. tenella* showed the most rapid increase. For illustrative purposes, models are extrapolated outside of the source data range in this figure. Data points for calibrating models of severe infection of *E. maxima* and *E. tenella* were not found in the literature, due to high levels of mortality. Within the simulations, no additional mortality was observed within the flock simulation models at the oocyst seed levels tested.

**Figure 4:**
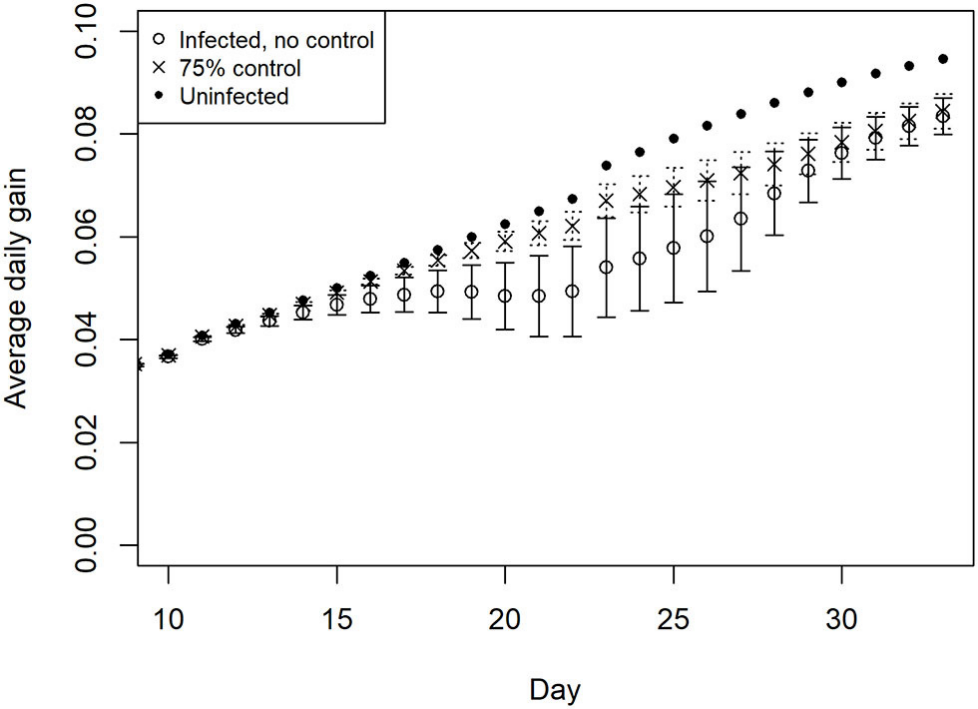
Impact of multiple *Eimeria* species (*E. acervulina, maxima, tenella*) on average daily weight gain in simulated infected broiler flocks, with 95% distribution, with and without control.

The average daily weight gain of chickens within the simulated flocks for uninfected and infected status is illustrated in Figure 4. Growth begins to deviate from the expected trajectory after ~2 weeks in the absence of control measures. The impact flattens growth for ~7 days. There is also a notable increase in variation between chickens at this stage of the production cycle. With the addition of control, illustrated here at 75% efficacy, the flattening of growth is significantly less pronounced than in the absence of control, nevertheless a divergence from uninfected flock growth rates is predicted.

**Figure 5:**
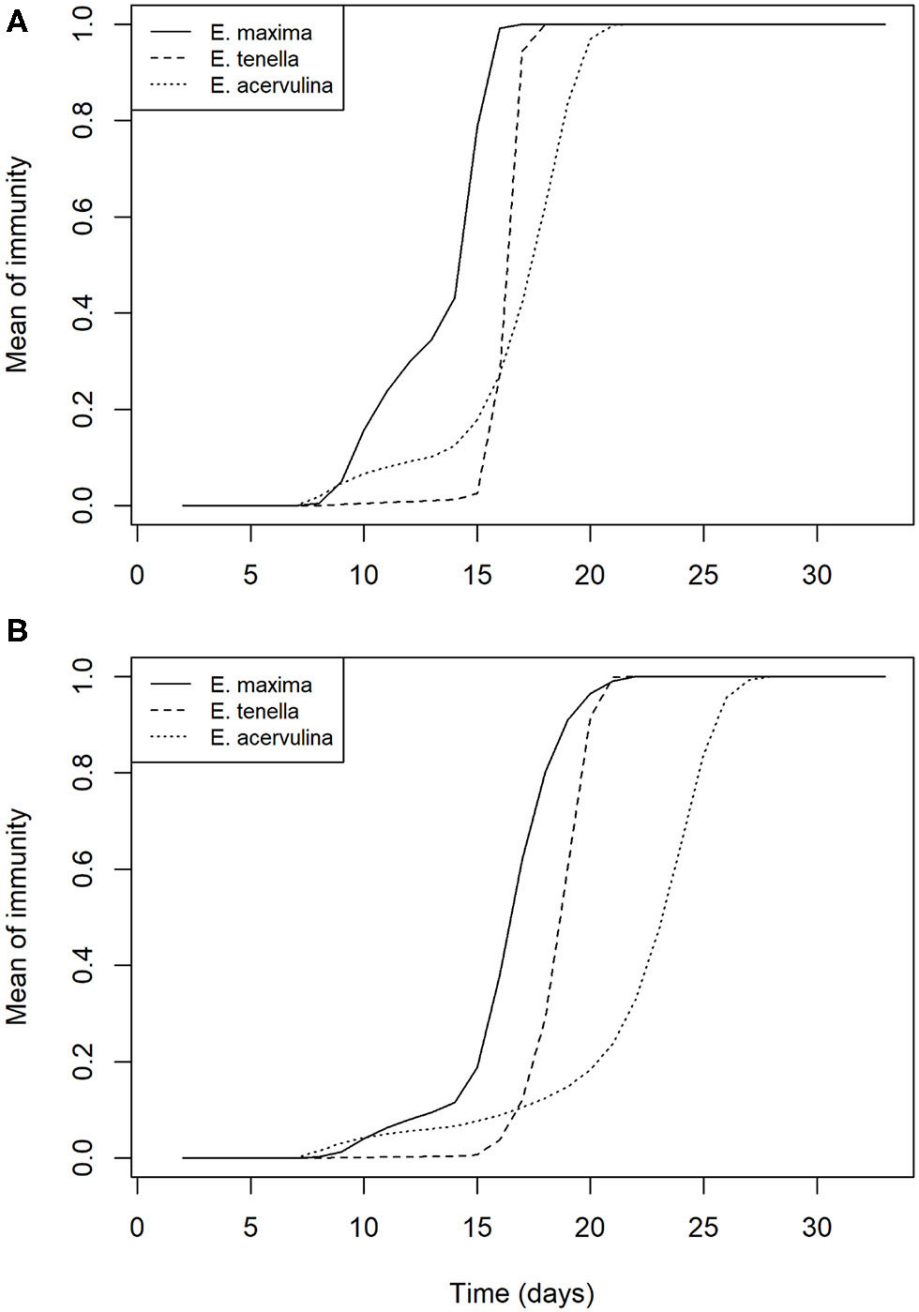
Mean within flock protective immunity against *Eimeria* species **(A)** in the absence of control measures; and **(B)** with control of *Eimeria* at 75% efficacy.

In the absence of control, full protective immunity to all three species is developed by day 20 on average. In the presence of increasing control efficacy, this development is slowed but not prevented, such that full protective immunity is delayed by an average of ~7 days for all species at 75% efficacy of control (Figure 5).

**Figure 6:**
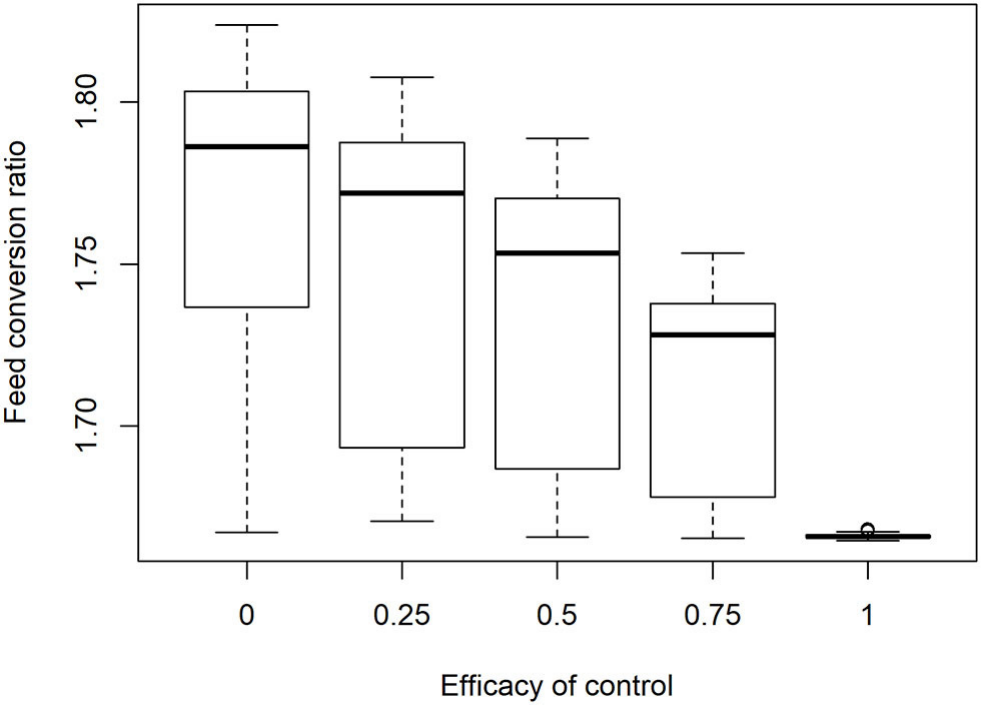
Impact of increasing control efficacy and variable infection pressure on efficiency of feed conversion in 350 simulated commercial broiler flocks.

Figure 6 illustrates the effect of infection and control on the efficiency of feed conversion in broiler flocks. This variation in results is generated by the variation in starting oocyst concentration and species present in each of the 350 simulations. The variation in outcomes across flocks was reduced with increasing efficacy of control. Between flock variation was extremely low when repeated model runs were made with equal initial oocyst concentrations at commercial flock sizes and stocking densities.

**Figure 7:**
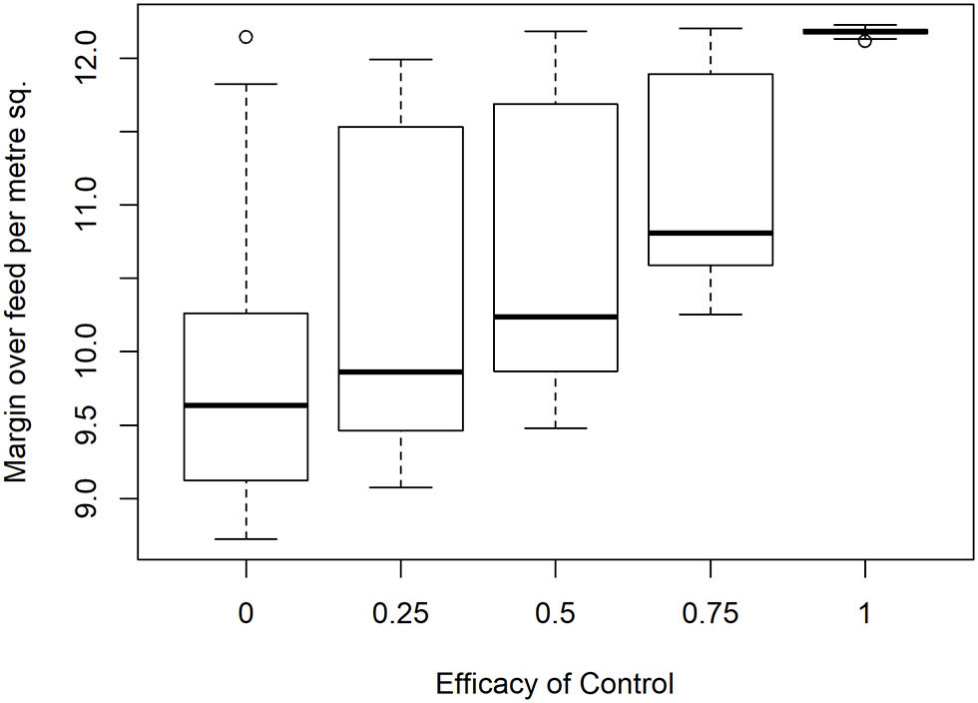
Impact of *Eimeria* infection on producer margin over feed costs (€/m^2^) under increasing levels of control efficacy and infection pressure in 350 simulated flocks.

To analyze the economic impacts of *Eimeria* and its control, a range of prices were applied to feed and output. Particular attention was given to the lowest margin when feed costs are high, output prices low and financial risks to producers are therefore greatest, it was found that median margins of €12.18 per m^2^ in the absence of disease are reduced to €9.63 at the median when control is completely ineffective, for a loss of €2.55 m^−2^ (Figure 7). A minimum of the margin distribution at <€9 m^−2^ was recorded in the total absence of control. Proportionally large changes were evident with relatively small changes in efficacy. The median impact of coccidiosis and changes in control efficacy across price ranges is presented in Table 5.

**Table 5:** Simulated changes in median producer margins over feed costs with varying price conditions and coccidiosis control efficacy in a typical European intensive broiler system.

		**Median change in margin over feed (**€**/m**^****2****^ **per flock)**				
		**Efficacy of control**				
**Feed price**	**Output price**	**100%**	**75%**	**50%**	**25%**	**0%**
Low	High	0	−1.58	−2.25	−2.67	−2.97
Median	Median	0	−1.47	−2.10	−2.49	−2.75
High	Low	0	−1.37	−1.94	−2.32	−2.55

## Discussion

This paper presents a first iteration of a model for coccidiosis impact in intensive broiler production including multiple species of *Eimeria*. The three species are those most commonly associated with intensive broiler systems, and the impact shown in the model suggests that *Eimeria* species remain a significant source of financial loss even in the presence of typical control measures. The economic analysis demonstrated the potential for coccidiosis to cause further significant losses in the absence of control. Even at a relatively efficacious control level of 75%, the average loss across the range of infection pressures measured is approaching €1.50 per m^2^ per flock.

At present, these results make are not considered representative of any particular national production system, since no assumptions were made with regard prevalence of each species, which could vary geographically, or initial oocyst concentration maintained within broiler houses after cleaning and disinfection protocols. These figures would need to be estimated for a stochastic simulation to look at national or regional losses to coccidiosis.

The search for data on control efficacy through EFSA found field isolates of *Eimeria* species with significantly higher levels of resistance, reducing control efficacy below 50%. An efficacy of 75% would have placed at the upper limit of the EFSA figures. The non-linear increasing rate of loss as control efficiency decreases is an important consideration when any change in management where marginal decreases in control efficacy could result.

The current political and societal climate is such that in the near term, changes in available coccidiosis control options are possible as pressure mounts on the continuing use of antimicrobial products in livestock agriculture. The results presented here serve to illustrate the challenges that must be considered when new control measures are proposed as a replacement for ionophores. Producer margins are sensitive to relatively small changes in control efficacy.

This assessment of the cost of *Eimeria* infection is not complete however. Revenue foregone due to delays to production, the costs of reactive treatment and secondary infections and the cost of cleaning and disinfection procedures between flocks are not estimated. The model ends the production cycle on the planned day and harvests chickens at substandard weight, quantifying change in output as lost mass. In reality, producers could extend the production cycle, which alters the dynamic of production in terms of output per year and the spreading of fixed costs. Additionally, any medications applied in a reactive manner to severe cases are not accounted for or costed in the model, although through discussions with producers across Europe it was established that this is relatively common course of action. A further analysis of lesion score data from routine monitoring of poultry flocks would be a way to establish the criteria under which additional control products are applied. This would allow a management-simulating component, based on the behavior of the producer, to be designed.

The uncertainty in feed price represents fluctuations in the market price. While it is acknowledged that some seasonal fluctuations in pricing are predictable and producers can hedge against this by forward purchasing, the proportion of farms entering into such arrangements or the impact of these arrangements on the distribution of feed prices received by producers as a whole is not known. The lower margin, where feed prices are high and output prices low, was then analyzed as the condition of greatest financial risk for poultry producers. The model could be further adapted to include other variable input and output prices and fixed costs to produce detailed analysis of farm budgets.

A modeling approach to analyzing policy change can mitigate the need for *in vivo* studies with consequent risks to animal welfare. Access to larger and more comprehensive field datasets would allow a more robust approach to validating model outcomes where some parameters are approximations of secondary data. This is particularly relevant when the data used to define critical parameters such as effects on growth rate are derived from single *Eimeria* lines, or from genetic lines of chicken that are no longer farmed commercially.

The model output suggests *Eimeria acervulina* may be considered the most significant of the three species studied, possibly on account of its high prevalence and lower immunogenicity giving it a longer duration of action within the flock. A caveat would be that between-strain variation within each species is not considered here. Further data on field strains and the range of pathogenic outcomes displayed would help define what could be considered a typical infection within the model. The model of coccidiosis impact is informed by available literature data which quantified dose dependent responses. The limit of these data is evidenced by the number of comparison points in Figure 3. In reality, this represents a simplified view of the field situation where variations in pathogenicity and immunogenicity of different strains of *Eimeria* species and host genetics could interact to produce a wider range of potential outcomes. Across repeated model runs with the same initial settings, the flock level results were found to be extremely consistent. This is a product of the homogenous-mixing mode of environmental contamination, such that oocysts are spread evenly per meter-squared. Where the first infected birds in the production cycle produce thousands or hundreds of thousands of new oocysts, this model quickly produces similar infection patterns when flocks are of typical commercial size.

Individual chicken-level variation in response is expected (63), and can be included within the model framework. Studies such as Hamzic et al. (53) have sought to quantify bird-level variation as part of genome-wide association studies. These types of data, on large samples of modern commercial lines of broilers could be invaluable in developing models with a greater level of resolution.

The model is designed to allow successive flock placements, with associated cleaning and downtime periods, although these have not yet been parameterized. The relationship between contamination level at slaughter date, the use of ionophores to control oocyst production, downtime cleaning and disinfection protocols, the development of ionophore and other drug resistances, and the initial contamination level at the start of the next flock cycle has not yet been established within the model. With appropriate data, this should be feasible and indeed desirable. While the current simulations show economic benefits in the use of ionophores, benefits may diminish significantly unless shuttle and rotation systems are employed, and this could be an important consideration when alternative control methods are investigated in isolation.

Data on flock-to-flock carry-over of *Eimeria* oocysts are difficult to find in the public domain. Indeed, data on oocyst numbers in the environment over the course of the flock cycle are difficult to find for European systems. Some data are available for US systems [e.g., Chapman et al. (64)] which operate on deep litter, but these are not applicable when cleaning and disinfection occurs between each flock.

Within the model simulations, each flock was seeded with a relatively low initial environmental oocyst dose. The step-wise increase of oocyst concentration in the environment and the dose-dependent response curves defined meant that by the time sufficiently large quantities of oocysts were available to constitute potentially lethal doses, chickens had developed partial immunity through prior exposure. While mortality as a direct result of *Eimeria* infection has been discussed, the frequency with which this occurs in the field, and whether it is successfully disaggregated causally from other forms of dysbiosis for which *Eimeria* infection is a known risk-factor, is not well-documented. Further attention should also be paid to the speed of immunity development to each species, particularly *Eimeria tenella*. It may be the case that a more complex model of the dynamics of immunity is required to reflect differences between each species.

As a potentiator of secondary infection, coccidiosis is particularly associated with necrotic enteritis caused by *Clostridium perfringens*. This effect was not quantified within the model as the complexity of environmental and dietary factors in the etiology of this disease would necessitate significant assumptions to be made to avoid a large increase in computational load. It is a potential future development of this model.

Experimental studies have shown that coinfection with multiple species of *Eimeria* can result in modifications to pathology when compared to a single-species infection, however the exact nature of these modifications depends on the specific *Eimeria* species in question and for several species is not documented. During co-infection with two species (*E. praecox and E. acervulina*) Répérant et al. (65) show an additive effect on growth and FCR, with coinfection producing greater change than single species infection. Conversely, Jenkins et al. (66) show multiple species infection having a protective effect. As a proportion of non-infected controls, 48% of healthy weight gain was observed during *E. maxima* infection, 90% weight gain during *E. praecox* infection, and 79% during coinfection. From the perspective of pathogen reproduction, Williams (67) demonstrated a reduction in reproductive capacity of co-infecting species when *E. acervulina* infection occurs concurrently with any of four other species (*E. tenella, E. maxima, E. brunetti, E. necatrix*). This could be attributed to the broad effects of the innate immune response, and a central role for multiple cytokines including interferon gamma in the response to different *Eimeria* species (68). Humoral immune responses, however, have been shown to be independent and specific for different *Eimeria* species such that coinfection does not produce a synergistic or competitive effect on immunity (69). Resolving this relationship is likely to be dependent on timing, strain, dose, and host immune response and therefore is likely to require large amounts of field data, or significant *in vivo* studies with the welfare and ethical considerations that that would raise. As a result, within this study a simple additive effect was incorporated, with the acknowledged limitation that this could result in overestimation of the impact of infection in multispecies cases.

In summary, the further development of this model would allow analysis of policy-relevant questions with respect to broiler production in intensive systems. The current model output illustrates the continuing sensitivity of producer margins to changes coccidiosis burden and control efficacy in broiler systems. This must be considered in any future changes in production standards or legislation.

## Data Availability Statement

The original contributions generated for the study are included in the article/supplementary material, further inquiries can be directed to the corresponding author.

## Author Contributions

DB, FT, and WG: conceptual work on *Eimeria* biology. WG, CB, and JR: conceptual work on modeling method. CB and JR: data collection with poultry producers. WG, CB, DB, FT, and JR: manuscript contribution. WG: model programming and analysis. All authors contributed to the article and approved the submitted version.

## Conflict of Interest

The authors declare that the research was conducted in the absence of any commercial or financial relationships that could be construed as a potential conflict of interest.
